# Examining the Impact of a Mobile Health App on Functional Movement and Physical Fitness: Pilot Pragmatic Randomized Controlled Trial

**DOI:** 10.2196/24076

**Published:** 2021-05-28

**Authors:** Matthew Jordan Stork, Ethan Gordon Bell, Mary Elizabeth Jung

**Affiliations:** 1 School of Health and Exercise Sciences The University of British Columbia Kelowna, BC Canada

**Keywords:** mHealth, functional movement, flexibility, strength, cardiovascular fitness

## Abstract

**Background:**

Numerous mobile apps available for download are geared toward health and fitness; however, limited research has evaluated the real-world effectiveness of such apps. The movr app is a mobile health app designed to enhance physical functioning by prescribing functional movement training based on individualized movement assessments. The influence of the movr app on functional movement and physical fitness (flexibility, strength, and cardiovascular fitness) has not yet been established empirically.

**Objective:**

This study aims to examine the real-world impact of the movr app on functional movement, flexibility, strength, and cardiovascular fitness.

**Methods:**

A total of 48 healthy adults (24 women and 24 men; mean age 24, SD 5 years) completed an 8-week pilot pragmatic randomized controlled trial in which they were randomly assigned to either 8-week use of the movr app (n=24) or 8-week waitlist control (n=24). Measures of functional movement (Functional Movement Screen [FMS]), strength (push-ups, handgrip strength, and countermovement jump), flexibility (shoulder flexibility, sit and reach, active straight leg raise [ASLR], and half-kneeling dorsiflexion), and cardiovascular fitness (maximal oxygen uptake [
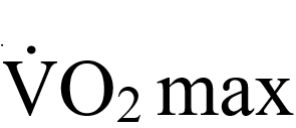
]) were collected at baseline and the 8-week follow-up.

**Results:**

Repeated measures analyses of variance revealed significant group-by-time interactions for the 100-point FMS (*P*<.001), shoulder flexibility (*P*=.01), ASLR (*P*=.001), half-kneeling dorsiflexion (*P*<.001), and push-up tests (*P*=.03). Pairwise comparisons showed that FMS scores increased from pre- to postintervention for those in the movr group (*P*<.001) and significantly decreased for those in the control group (*P*=.04). For shoulder flexibility, ASLR, half-kneeling dorsiflexion, and push-up tests, improvements from pre- to postintervention were found in the movr group (all values of *P*<.05) but not in the control group (all values of *P*>.05). There were no changes in the sit and reach or handgrip strength test scores for either group (all values of *P*>.05). A significant main effect of time was found for the countermovement jump (*P*=.02), such that scores decreased from pre- to postintervention in the control group (*P*=.02) but not in the movr group (*P*=.38). Finally, a significant group-by-time interaction was found for 
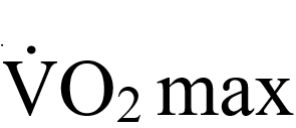
 (*P*=.001), revealing that scores decreased pre- to postintervention in the control group (*P*<.001), but not in the movr group (*P*=.54).

**Conclusions:**

The findings revealed that movr improved indices of functional movement (FMS), flexibility (shoulder, ASLR, and dorsiflexion), and muscular endurance (push-ups) over an 8-week period compared with the control group while maintaining handgrip strength, lower body power (countermovement jump), and cardiovascular fitness (
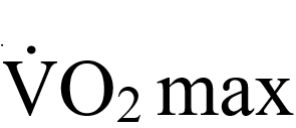
). Thus, this study provides initial evidence of the effectiveness of the movr app for enhancing functional movement and physical fitness among healthy adults.

**Trial Registration:**

ClinicalTrials.gov NCT04865666; https://clinicaltrials.gov/ct2/show/NCT04865666

## Introduction

### Background

Numerous mobile apps are available for download that are geared toward health and fitness [1]. Unfortunately, most of these apps are not evidence based and have been deemed to be ineffective [1-4]. Furthermore, limited research evaluating the real-world effectiveness of such apps exists [4]. In a review of the literature on mobile apps used in health interventions, the authors concluded that “the potential for scalable behavioral interventions through these technologies is promising, but largely untapped...researchers should focus on conducting rigorous RCT (randomized controlled trial) studies with adequately powered sample sizes to determine the utility of app-based health interventions” [1]. Others have echoed this notion by highlighting that although technology-based interventions (eg, those that use mobile technology) have the advantage of being cost-effective, convenient, and accessible, there is a need for RCTs that examine the efficacy of such interventions [5].

Although many health and fitness apps are designed to promote increased physical activity or exercise participation [6], very few apps are designed to enhance the quality of functional movement and physical fitness while catering to individual needs. movr is a mobile health (mHealth) app that takes a personalized approach to improve user flexibility, strength, and overall fitness by prescribing functional movement training based on a user’s own movement assessments. This is meaningful because physical fitness components of strength, flexibility, and stability have been linked to health, musculoskeletal injury risk, injury treatment, and performance of activities of daily living [7]. Furthermore, improving people’s physical fitness and the quality of their functional movement may subsequently help facilitate further physical activity behavior. Physical fitness and physical activity are both important for health outcomes [8], and improved fitness has been associated with improved quality of life [9].

movr uses self-reported movement assessments to identify movement deficiencies and prescribes exercises to target those deficiencies. Other user information, such as time and equipment available and desired exercise focus, is also accounted for when generating personalized workout sessions. However, the real-world impact of movr has not yet been evaluated. Before the broad uptake of this newly developed app is encouraged, it is imperative to test its utility and, in particular, its potential to influence functional movement and physical fitness.

The Functional Movement Screen (FMS) is a cost-effective tool developed to assess functional movement based on seven fundamental movement patterns [10]. A large body of research literature has investigated the use of the FMS for predicting the future risk of injury (eg, [11,12]). Given the movr app’s goal of enhancing people’s quality of movement, the FMS is a measurement tool that is well suited to test its effectiveness in improving individual functional movement capacity. A limitation of the FMS is that it is typically scored on a 0-3 scale (21-point system), and it has been suggested that this scaling system may not be overly sensitive to changes over time following several weeks of movement-based training (eg, [13,14]). For example, Bodden et al [15] highlighted that based on the 0-3 scoring criteria, there were broad ranges of movement quality even when participants were assigned the same score of 2 on a given movement (ie, some scores of 2 had better movement quality than others). Notwithstanding, Butler et al [13] introduced a more detailed criterion that can be reliably used to assess the FMS movements using a 100-point scale system, and Frost et al [14] have since adapted further subcriteria to the 100-point scale and have considered it to be a *research standard* version of the FMS. To our knowledge, the FMS and the physical fitness assessments administered in this study have never been used to examine the effectiveness of a mobile app for improving physical functioning.

Owing to the COVID-19 global pandemic, there has been a worldwide risk of people spending more time being sedentary in their homes and less time being physically active [16,17]. Unfortunately, these trends may persist in the foreseeable future. mHealth apps such as movr offer remote options for people to assess their functional movement in real time, work on their physical fitness, and move more frequently without the need for extensive exercise resources. Thus, there is potential for movr to not only enhance functional movement and physical fitness but also to do so in a digital format that is accessible in a time of physical and social distancing.

### Purpose and Hypotheses

This study aims to examine the real-world impact of movr on functional movement, flexibility, strength, and cardiovascular fitness (maximal oxygen uptake [
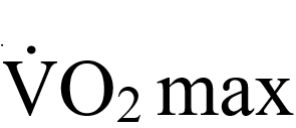
]) in a sample of healthy women and men. Given the aims of movr, it was hypothesized that from baseline to 8-week follow-up, participants in the movr group would experience improvements in functional movement, flexibility, and muscular endurance (push-ups) compared with those in the waitlist control group (*H*_1_). Considering the movr app’s primary focus on functional movement and limited focus on building aerobic fitness, grip strength, or explosive movement, it was hypothesized that 
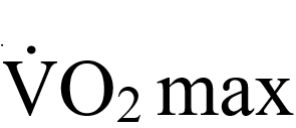
, handgrip strength, and lower body power (vertical jump) would be maintained (no increase or decrease) over the 8-week intervention period for both the movr and control groups (*H*_2_). To our knowledge, this is the first RCT study to evaluate the effects of an mHealth app on multiple indices of physical fitness and functioning, including functional movement screening.

## Methods

### Study Design Overview

This study consisted of baseline testing (visit 1) and an 8-week pilot pragmatic RCT [18]. This was designed with a pragmatic intent, as it was conducted in a real-world context under the usual circumstances [19,20]. Participants were randomly assigned to either an 8-week use of the movr app or 8-week waitlist control. Randomization was stratified by self-identified gender and was completed using a random number generator. Following the 8-week intervention, participants returned to the laboratory for follow-up testing (visit 2). The first wave of 24 participants (12 movr and 12 control) completed visit 1 of the study in September and October 2019 and visit 2 in November and December 2019, whereas the second wave of 24 participants (12 movr and 12 control) completed visit 1 in November and December 2019 and visit 2 in January and February 2020.

### Participants

Considering the pilot objectives of this pragmatic RCT [21] and following recommendations for calculating the sample size for such designs [22,23], a sample of 40 participants (n=20 per group) was sought. Accounting for a 20% loss to follow-up, we recruited a sample size of 50 participants (n=25 per group). Eligible participants were healthy men and women aged between 18 and 50 years who had the ability to read and write English and owned a mobile phone that could download apps from the Apple App Store or Google Play Store. Participants were excluded from the study if they had previously used the movr app or had any contraindications to exercise based on the Get Active Questionnaire. The University of British Columbia Clinical Research Ethics Board approved the study protocol, and participants were recruited through word of mouth (ie, members of the research team and laboratory group provided general study information to individuals who may have been interested in participating) and study advertisements (ie, poster advertisements on campus, sign-up sheets in classes, and via social media outlets such as Facebook). All participants provided written informed consent and received a Can $50 (US $37.79) gift card on completion of the study.

### Functional Movement

The 100-point version of the FMS [13,14] was used to identify individual movement patterns at baseline and to detect any changes in mobility and stability following the 8-week intervention. The FMS is a valid and reliable screening tool for assessing whole body movement patterns [24,25] and consists of seven core movement tests (deep squat, hurdle step, inline lunge, active straight leg raise [ASLR], shoulder flexibility, trunk stability push-up, and quadruped rotary stability). The seven FMS movement screens were performed by each participant and recorded using a portable observation laboratory (Noldus) with two video cameras (one recording in the sagittal plane [side] view and the other in the frontal plane [front or back] view). The standardized FMS verbal instructions were provided by one researcher, whereas the other researcher ensured that the camera angles captured all movements. The video-based testing was completed according to the same protocols as Butler et al [13] to minimize participant burden during laboratory visits and to blind participants to their FMS scores for each of the movements (ie, the researchers did not score the movements with participants present). Scoring of the FMS was completed at a later time using the video recordings.

The first (MJS) and second (EGB) authors scored the FMS tests of the first 10 participants independently and then reached consensus using the 100-point criteria provided by Butler et al [13] and the research standard version adapted by Frost et al [14]. This process of FMS scoring of the first 10 participants was used to help clarify the use of the 100-point scoring criteria and inform the scoring of all remaining participants. EGB provided the initial scoring of all remaining participants, which was then verified by MJS. Any discrepancies were addressed, and full consensus was achieved between both authors on the final scoring.

### Physical Fitness

Multimedia Appendix 1 provides full details of the procedures followed for the flexibility, muscular endurance, handgrip strength, and lower body power tests, and Multimedia Appendix 2 provides sample images of such tests.

#### Flexibility Tests

The shoulder reach flexibility test was used to assess upper body flexibility, and the sit and reach, ASLR, and the half-kneeling dorsiflexion tests were used to assess lower body flexibility [26,27]. For the shoulder reach test, a soft tape measure was used to measure the distance between the participants’ closed fists, and for the ASLR, the range of motion in degrees was measured on each leg using a digital inclinometer (Metriks). For each of the flexibility tests (excluding sit and reach), scores from the left and right sides (arm or leg) were summed, and the total scores were reported. A lower measurement score on the shoulder flexibility test indicates greater flexibility, whereas higher scores on the sit and reach, ASLR, and half-kneeling dorsiflexion tests indicate greater flexibility.

#### Muscular Endurance

A push-up test was used to determine the maximal number of successive push-up repetitions that participants could complete until failure. The test protocols followed the Canadian Society for Exercise Physiology recommendations [28], including the use of a modified push-up protocol for all women in the study.

#### Handgrip Strength

Handgrip strength [29] was assessed using a maximal voluntary contraction of an isometric handgrip squeeze using a Smedley spring handgrip dynamometer (BASELINE). Scores from the left and right hands were summed, and the total scores were reported.

#### Lower Body Power

A countermovement jump was conducted using the My Jump 2 mobile app on an iPhone 8 to assess lower body power. The validity and reliability of the My Jump 2 app has been established previously [30].

#### Cardiovascular Fitness

Participants performed an incremental 
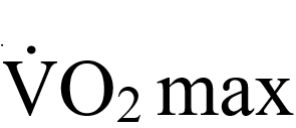
 test on a cycle ergometer (Lode Excalibur Sport) as described previously (eg, Gillen et al [31]). The resistance on the cycle ergometer was automatically increased (1 W every 3 s for women and 1 W every 2 s for men) until participants reached volitional exhaustion or could no longer maintain a pedal cadence of at least 50 rpm. A metabolic cart with an automated gas collection system (Parvo Medics, TrueOne 2400) was used to continuously collect expired gas samples, and 
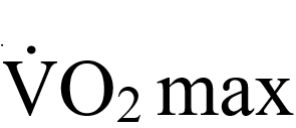
 was calculated using the mean of the highest average oxygen consumption over a 30-second period (in mL/kg/min). Peak power output in watts and maximal heart rate were also measured during each 
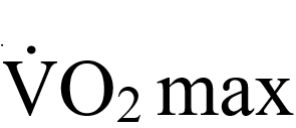
 test.

### Protocol

#### Baseline Testing (Visit 1)

Eligible participants read the consent form and provided informed consent. Participants then completed a baseline demographic questionnaire and self-reported their physical activity levels using the International Physical Activity Questionnaire—Short Form on a laboratory computer. Participants’ height and body mass were then assessed using a stadiometer (Seca 700). The order of functional and fitness testing was standardized for consistency across all visits and participants and to minimize the effects of fatigue on subsequent tests. Participants started with a light warm-up consisting of 2 minutes of continuous pedaling on a cycle ergometer, followed by testing in the following order: anthropometric measures, sit and reach test, half-kneeling dorsiflexion test, handgrip strength test, countermovement jump, push-up test, seven FMS movements (including shoulder reach and ASLR tests), and 
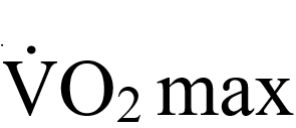
 test.

Before leaving the laboratory, participants in the movr group were asked to download the movr app on their mobile phones. They were then assisted in creating a unique movr account using their assigned participant ID code. The accounts were dummy accounts (with unique ID codes as names) to ensure that participants did not use their personal information. This protocol was also used to track and retrieve the participant app usage data from the movr database server.

#### Waitlist Control Group

Participants in the control group were instructed to maintain their usual physical activity, diet, and sleep behavior [20,32,33] for the next 8 weeks and to avoid any specialized exercise training during this period. An attention control group was avoided because of the pragmatic nature of this study and several cited issues with attention control in behavioral interventions (eg, equal attention between groups does not necessarily eliminate unintended differences between groups and attention control groups can lead to inadvertent interventions [32,33]). Following the 8-week study period, individuals in the control group were permitted to download and use the movr app if they chose to.

#### Movr Group

Participants in the movr group were also instructed to maintain their usual physical activity, diet, and sleep behavior for the next 8 weeks and to avoid any specialized exercise training during this period, but they were also asked to use the movr app to supplement their current activity. Within 24 hours after their first laboratory visit, participants were instructed to complete the 10 self-reported *Movement Assessment* tests through their individual movr app account. These movement tests are performed to assess mobility, motor control, and strength and are used to determine deficiencies in movement patterns and to provide prescriptive information for exercise selection within the app. Participants were prompted (via email) to complete their Movement Assessments again at 4 and 8 weeks.

The exercises prescribed through the movr app were accompanied with videos, images, and detailed instructions on how to complete them. These exercises consisted of basic movement and mobility patterns and were designed to promote functional movement. Participants were instructed to complete a total of four *Minis* and two workout *Builder* sessions per week. *Minis* consist of 5-minute sessions designed to improve participant movement capacity (ie, flexibility, motor control, and muscular strength) and are intended to be easily incorporated into an individual’s daily life. There are two forms of Minis available on the app: (1) *Your Minis*, which are designed to address specific areas for improvement based on a user’s most recent Movement Assessment scores and (2) *Everyday Minis*, which can be used for a variety of everyday situations (eg, taking a desk break or pre- or postexercise). Specifically, participants were asked to complete a minimum of two Your Minis out of the four total Minis to be completed per week. Workout *Builders* are designed to be longer exercise sessions tailored to the user’s desired exercise time (15, 30, 45, or 60 min), equipment available (TRX band, kettlebell, chin-up bar, dumbbells, foam roller, or none), target body region (lower, upper, or whole body), and specific exercise goal (*get sweaty*, *build strength*, or *develop mobility*). Version 3.6 of the movr app was used at the start of data collection and was updated to version 4.1 over the course of the trial. No significant changes were made to the app or exercise prescription functionality throughout the updates.

#### Follow-up Testing (Visit 2)

Eight weeks later, participants were asked to return to the laboratory to complete follow-up self-reported physical activity and functional and fitness testing. The testing order and procedures were completed exactly as they were at visit 1.

### Statistical Analyses

Separate 2 (group)×2 (time) mixed repeated measures (RM) analysis of variance (ANOVA) was conducted on the functional and physical fitness outcomes to examine between-group (movr vs control) and within-group (pre- to postintervention) differences. A mixed RM multivariate analysis of variance (MANOVA) was used for outcome variables that were conceptually intercorrelated [34], and significant *F* tests were followed by subsequent mixed RM ANOVAs. For all tests, significant effects were followed by Bonferroni-corrected pairwise comparisons to detect between- and within-group differences. The magnitude of the observed effect sizes was reported as partial eta squared (η_p_^2^). All analyses were conducted using SPSS version 26, and significance was set at an α level of *P*<.05.

## Results

### Participants

One man in the movr group did not show up for follow-up testing, and one woman in the control group dropped out of the study for reasons unrelated to the study. Thus, a total of 48 participants (24 women and 24 men) completed the study (movr=24 and control=24), and their characteristics are presented in Table 1. One woman was unable to complete the push-up test at either visit due to a chronic shoulder injury, so these data were considered as missing and were not included in the push-up test analyses.

**Table 1:** Participant baseline characteristics.

Variable			movr (n=24)		Control (n=24)
Age (years), mean (SD)			22.9 (5.3)		24.3 (5.3)
Body mass (kg), mean (SD)			71.2 (14.3)		70.5 (11.0)
Height (cm), mean (SD)			171.4 (11.6)		171.2 (9.3)
BMI (kg/m^2^), mean (SD)			24.1 (3.5)		23.9 (2.0)
Waist circumference (cm), mean (SD)			77.9 (9.2)		78.5 (7.1)
Maximal heart rate (bpm), mean (SD)			187.6 (8.7)		187.5 (12.0)
Peak power output (W), mean (SD)			270.9 (83.5)		285.0 (81.9)
Maximal oxygen uptake (mL/kg/min), mean (SD)			40.4 (9.5)		43.1 (9.3)
Moderate- to vigorous-intensity physical activity (metabolic equivalents min per week), mean (SD)			2520.7 (1742.6)^a^		2494.2 (1417.5)
**Gender, n (%)**					
	Women	12 (50)		12 (50)	
	Men	12 (50)		12 (50)	
**Sex, n (%)**					
	Female	12 (50)		12 (50)	
	Male	12 (50)		12 (50)	
**Race, n (%)**					
	White	18 (75)		22 (92)	
	Indigenous	0 (0)		1 (4)	
	Chinese	1 (4)		0 (0)	
	Southeast Asian	2 (8)		0 (0)	
	South Asian	1 (4)		0 (0)	
	Latin American	1 (4)		1 (4)	
	West Indian	1 (4)		0 (0)	
**Highest education** **, n (%)**					
	High school	11 (46)		12 (50)	
	Trades certificate or diploma	1 (4)		2 (8)	
	Nonuniversity certificate or diploma	3 (13)		0 (0)	
	University certificate or diploma	7 (29)		4 (17)	
	Postgraduate degree	2 (8)		6 (25)	
**Occupation, n (%)**					
	Working full time	1 (4)		3 (13)	
	Working part time	3 (13)		2 (8)	
	Student	18 (75)		19 (79)	
	Retired	1 (4)		0 (0)	
**Annual household income (Can $)** **, n (%)**					
	0-25,000 (US $0-US $18,895)	13 (54)		7 (29)	
	25,000-50,000 (US $18,895-US $37,790)	4 (17)		5 (21)	
	50,000-75,000 (US $37,790-US $56,685)	2 (8)		4 (17)	
	75,000-100,000 (US $56,685-US $75,580)	1 (4)		0 (0)	
	>100,000 (>US $75,580)	2 (8)		3 (13)	
	Prefer not to answer	2 (8)		5 (21)	

^a^n=23 for moderate- to vigorous-intensity physical activity (metabolic equivalents min per week) in the movr group only because of missing data.

### Functional Movement

**Figure 1:**
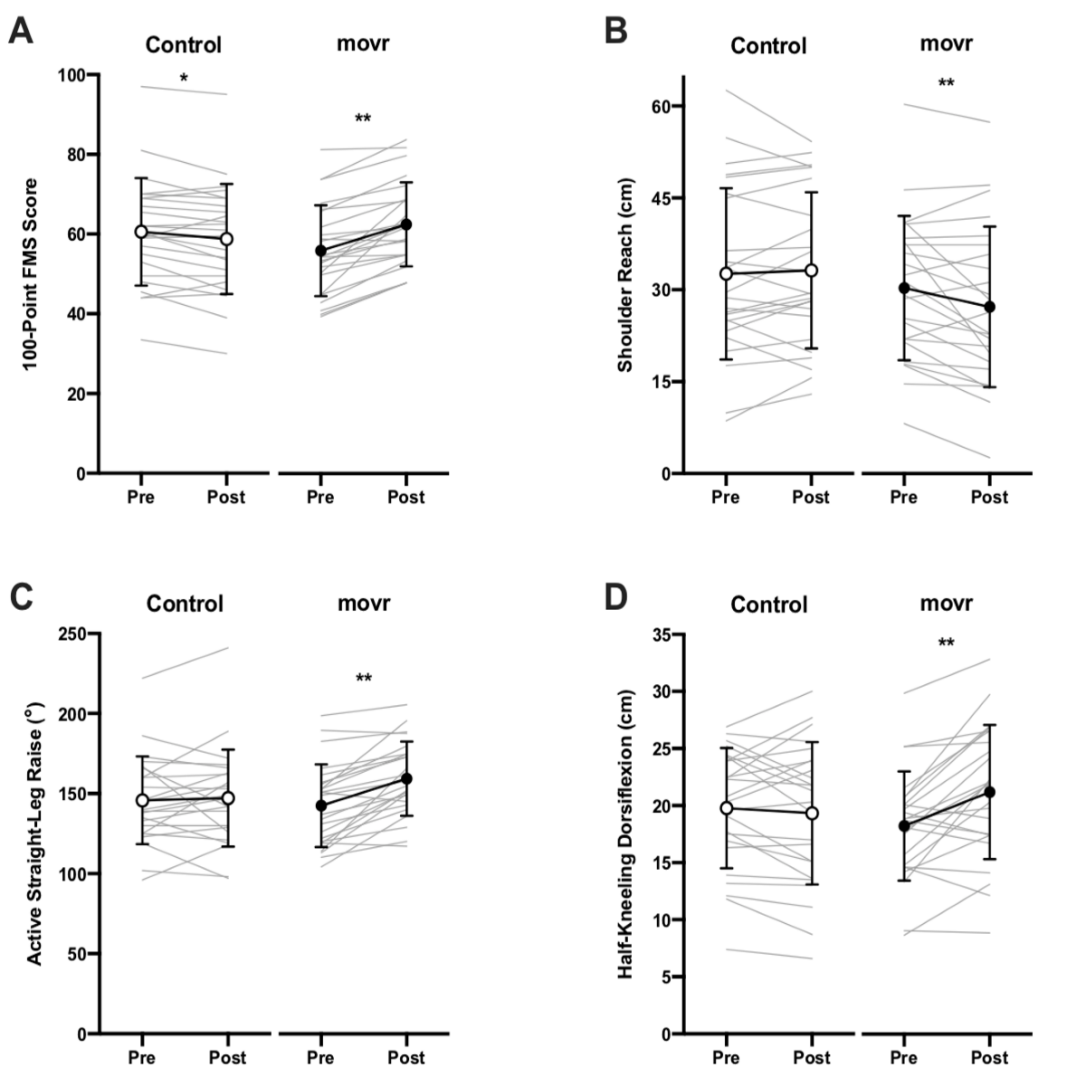
Measurements of (A) 100-point Functional Movement Screen, (B) shoulder reach, (C) active straight leg raise, and (D) half-kneeling dorsiflexion before (pre) and after (post) the 8-week intervention period. Circles with black connecting lines represent sample means with SD error bars, whereas gray lines represent individual participant data points. Asterisks indicate significant differences between pre- and postintervention values within a given group (*P<.05; **P<.01). FMS: Functional Movement Screen.

A 2×2 mixed RM ANOVA on 100-point FMS scores showed a significant group-by-time interaction (*F*_1,46_=47.55; *P*<.001; η_p_^2^=0.51). Pairwise comparisons revealed that FMS scores significantly increased from pre- to postintervention for those in the movr group (mean 56.08, SD 11.40 to mean 62.71, SD 10.56; *P*<.001) and significantly decreased for those in the control group (mean 60.60, SD 13.53, to mean 58.77, SD 13.81; *P*=.04; Figure 1). A breakdown of the mean scores for each of the seven different FMS movements is presented in Table 2.

**Table 2:** Mean values for the 100-point Functional Movement Screen scores pre- and postintervention.

Functional Movement Screen test	movr			Control			Maximum possible test score
	Preintervention (n=24), mean (SD)	Postintervention (n=24), mean (SD)	Preintervention (n=24), mean (SD)		Postintervention (n=24), mean (SD)		
Deep squat	7.00 (3.84)	9.00 (4.22)	7.83 (4.57)		7.96 (4.41)	18	
Hurdle step	14.38 (1.79)	14.67 (1.94)	14.98 (1.58)		14.35 (1.56)	18	
Inline lunge	12.25 (3.67)	14.21 (3.01)	13.71 (3.28)		12.96 (3.75)	20	
Shoulder mobility	6.67 (1.63)	6.92 (1.77)	6.33 (2.01)		6.17 (2.28)	8	
Active straight leg raise	6.75 (3.80)	7.92 (3.36)	6.08 (4.06)		5.92 (4.31)	12	
Trunk stability push-up	5.00 (4.03)	5.29 (4.24)	6.83 (4.60)		6.67 (4.56)	12	
Rotary stability	4.04 (1.12)	4.71 (1.73)	4.83 (1.95)		4.75 (1.85)	12	
*Composite score^a^*	*56.08 (11.40)*	*62.71 (10.56)^b^*	*60.60 (13.53)*		*58.77 (13.81)^c^*	*100*	

^a^Italicization is to indicate that it is the total of all previous rows.

^b^Significant differences between pre- and postintervention composite Functional Movement Screen scores within a given group (*P*<.01).

^c^Significant differences between pre- and postintervention composite Functional Movement Screen scores within a given group (*P*<.05).

### Physical Fitness

#### Flexibility Tests

A 2×2 RM MANOVA was computed across the four flexibility measures of shoulder flexibility, sit and reach, ASLR, and half-kneeling dorsiflexion. Using Pillai trace, we found that there was a significant omnibus group-by-time interaction (*V*=0.37; *F*_4,43_=6.38; *P*<.001; η_p_^2^=0.37).

#### Shoulder Flexibility

A 2×2 mixed RM ANOVA on the shoulder reach test showed a significant group-by-time interaction (*F*_1,46_=6.58; *P*=.01; η_p_^2^=0.13). Pairwise comparisons revealed that shoulder flexibility significantly improved (scores lowered) from pre- to postintervention for those in the movr group (mean 30.34, SD 11.80 cm, to mean 27.28, SD 13.15 cm; *P*=.003) but not for those in the control group (mean 32.63, SD 13.99 cm, to mean 33.16, SD 12.74 cm; *P*=.59; Figure 1).

#### Sit and Reach Test

A 2×2 mixed RM ANOVA on the sit and reach test showed no significant main effects or interaction effects (all values of *P*>.05).

#### ASLR Test

A 2×2 mixed RM ANOVA on the ASLR test showed a significant group-by-time interaction (*F*_1,46_=11.95; *P*=.001; η_p_^2^=0.21). Pairwise comparisons revealed that ASLR significantly improved (scores increased) from pre- to postintervention for those in the movr group (mean 143.46, SD 26.03 degrees, to mean 160.50, SD 23.36 degrees; *P*<.001) but not for those in the control group (mean 145.79, SD 27.39 degrees, to mean 147.13, SD 30.78 degrees; *P*=.68; Figure 1).

#### Half-Kneeling Dorsiflexion

A 2×2 mixed RM ANOVA on half-kneeling dorsiflexion showed a significant group-by-time interaction (*F*_1,46_=14.23; *P*<.001; η_p_^2^=0.24). Pairwise comparisons revealed that dorsiflexion significantly improved (scores increased) from pre- to postintervention for those in the movr group (mean 18.33, SD 4.81 cm, to mean 21.30, SD 5.91 cm; *P*<.001) but not for those in the control group (mean 19.77, SD 5.27 cm, to mean 19.32, SD 6.25 cm; *P*=.49; Figure 1).

#### Strength and Power Tests

A 2×2 RM MANOVA was computed across the three strength and power measures of push-ups, handgrip strength, and countermovement jump. Using Pillai trace, we found that there was a significant omnibus effect of time (*V*=0.19; *F*_3,43_=3.28; *P*=.03; η_p_^2^=0.19).

#### Push-up Test

**Figure 2:**
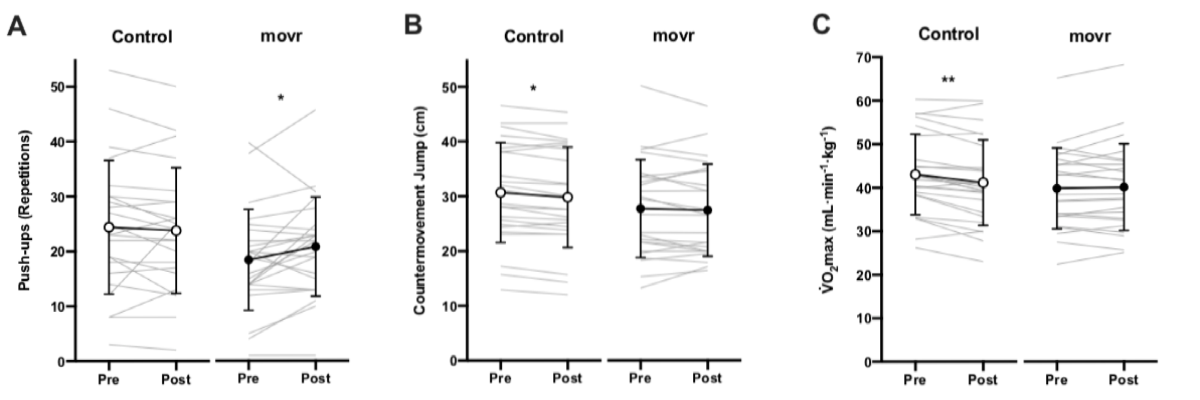
Measurements of (A) push-ups, (B) countermovement jump, and (C) maximal oxygen uptake (<inline-graphic xlink:href="mhealth_v9i5e24076_fig3.png" xlink:type="simple" mimetype="image"/>) before (pre) and after (post) the 8-week intervention period. Circles with black connecting lines represent sample means with SD error bars, whereas gray lines represent individual participant data points. Asterisks indicate significant differences between pre- and postintervention values within a given group (*P<.05; **P<.01). <inline-graphic xlink:href="mhealth_v9i5e24076_fig3.png" xlink:type="simple" mimetype="image"/>: maximal oxygen uptake.

A 2×2 mixed RM ANOVA on push-ups showed a significant group-by-time interaction (*F*_1,45_=5.06; *P*=.03; η_p_^2^=0.10). Pairwise comparisons revealed that push-ups significantly increased from pre- to postintervention for those in the movr group (mean 18.50, SD 9.27 repetitions, to mean 20.92, SD 9.09 repetitions; *P*=.01) but not for those in the control group (mean 24.39, SD 12.16 repetitions, to mean 23.78, SD 11.46 repetitions; *P*=.53; Figure 2).

#### Handgrip Strength

A 2×2 mixed RM ANOVA on handgrip strength showed no significant main effects or interaction effects (all values of *P*>.05).

#### Countermovement Jump

A 2×2 mixed RM ANOVA on countermovement jump showed only a significant main effect of time (*F*_1,46_=5.79; *P*=.02; η_p_^2^=0.11). None of the other main effects or interactions were significant (all values of *P*>.05). Pairwise comparisons for time revealed that countermovement jump significantly decreased from pre- to postintervention for those in the control group (mean 30.69, SD 9.11 cm, to mean 29.83, SD 9.19 cm; *P*=.02) but not for those in the movr group (mean 27.82, SD 8.97 cm, to mean 27.52, SD 8.43 cm; *P*=.38; Figure 2).

#### Cardiovascular Fitness

A 2×2 mixed RM ANOVA on 
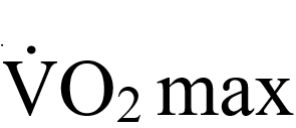
 showed a significant group-by-time interaction (*F*_1,46_=11.50; *P*=.001; η_p_^2^=0.20). Pairwise comparisons revealed that 
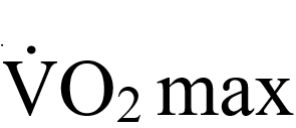
 significantly decreased from pre- to postintervention for those in the control group (mean 43.06, SD 9.27 mL/kg/min, to mean 41.16, SD 9.84 mL/kg/min; *P*<.001) but not for those in the movr group (mean 40.42, SD 9.46 mL/kg/min, to mean 40.70, SD 10.14 mL/kg/min; *P*=.54; Figure 2).

### Movr Usage Data

One woman in the movr group experienced technical issues related to the app that impacted her usage data being recorded on the movr database server. As such, her data were considered as missing and were not included in the movr usage reports. Over the 8-week study period, participants in the movr group completed an average of 2.20 (SD 1.27) Minis (mean 1.40, SD 0.83 Your Minis, and mean 0.80, SD 0.77 Everyday Minis) and an average of 0.96 (SD 0.72) Builders per week (n=23). In terms of total time spent completing sessions, participants in the movr group completed an average of 11.01 (SD 6.35) minutes per week completing Minis and an average of 17.93 (SD 14.68) minutes per week completing Builders, with a total average usage of 28.94 (SD 18.13) minutes per week (n=23). Note that the time spent completing Minis and Builders does not include the time participants spent completing the Movement Assessments.

## Discussion

### Principal Findings

The purpose of this study was to examine the real-world impact of movr on functional movement, strength, flexibility, and cardiovascular fitness. The main findings were that 8 weeks of using movr for an average of 29 minutes per week improved functional movement (FMS), most measures of flexibility (shoulder, ASLR, and dorsiflexion), and muscular endurance (push-ups) and led to the maintenance of handgrip strength, lower body power (countermovement jump), and cardiovascular fitness (
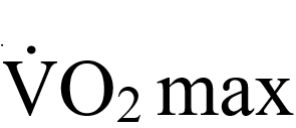
). These findings illustrate the potential real-world effectiveness of the movr app for enhancing physical functioning.

### Functional Movement

Consistent with *H*_1_, the 100-point FMS scores increased from pre- to postintervention for participants in the movr group compared with the control group. Interestingly, the FMS scores decreased over time for participants in the control group. Previous studies have also reported increases in FMS scores over the course of several weeks of specialized functional training or yoga [15,35,36]. In these studies, participant samples consisted of firefighters [35], American football players [36], and mixed martial arts athletes [15], and the duration of the interventions ranged from 6 to 8 weeks. Another study [14] failed to detect any changes in FMS scores following a 12-week functional training intervention among firefighters.

Notably, two of the abovementioned studies [35,36] did not include a control group in their design, three of these studies consisted of highly specialized and individualized training programs (including components that specifically targeted movement deficits identified by baseline FMS scores) [14,15,36], and all four studies included supervised training sessions that were led by trained exercise professionals (eg, strength and conditioning specialists). In addition, the participants in each of these studies were all highly trained individuals. We are unaware of any previous studies that demonstrated improvements in FMS scoring from pre- to postintervention that included training programs that were delivered entirely remotely and unsupervised or consisted of training programs that were not prescribed by exercise specialists. As such, the current findings demonstrate the potential for mHealth apps such as movr to enhance functional movement without requiring the supervision of professionals and among individuals who are not highly trained.

FMS has become a popular screening tool among researchers and exercise practitioners (eg, strength and conditioning coaches and physical therapists) and has been used to prescribe exercise and rehabilitation programs based on identified movement deficiencies (eg, dos Santos et al [11]). There has also been continued interest in using the FMS as an injury prevention tool that can be used to detect predisposition to injury and/or predict future injury [11,12,24]. In this sense, it is possible that the improvements in FMS scores found for the movr group may have meaningful implications for reducing the risk of injury. However, this postulation should be interpreted cautiously as findings from several systematic reviews and meta-analyses have drawn conflicting conclusions. Some have supported the predictive utility of the FMS for future injury (eg, dos Santos et al [11] and Bonazza et al [24]), whereas others do not support the use of FMS as an injury prediction tool (eg, Moran et al [12]) or suggest that the heterogeneity in studied sample populations makes it challenging to draw definitive conclusions [37]. Future research on this topic is required before the injury-related implications of the current FMS findings can be considered further.

### Flexibility Tests

In line with *H*_1_, measurements of shoulder flexibility, ASLR, and half-kneeling dorsiflexion showed an improvement from pre- to postintervention for participants in the movr group but not for participants in the control group. However, inconsistent with *H*_1_, sit and reach did not change over the course of the intervention period for either group. These findings suggest that the movr app was indeed effective for improving indices of upper and lower body flexibility, with the exception of the sit and reach test. Both shoulder flexibility and lower body flexibility are key areas that are identified via the Movement Assessments and targeted through the Minis and Builders sessions within the movr app.

Intriguingly, although the ASLR and sit and reach tests both measure components of hamstring flexibility, only ASLR showed a significant improvement for those who used movr. This may be explained, in part, by evidence that sit and reach test scores are strongly influenced by factors other than hamstring extensibility [38], such as pelvic tilt and lumbar spine flexion [39]. The sit and reach consists of passive hamstring lengthening, whereas the ASLR requires an individual to actively raise each leg under their control. Furthermore, abdominal wall bracing and lumbar stability are needed to minimize the risk of pain while performing the ASLR [40]. It may be that the movr app’s focus on improving core activation, strength, and motor control may have differentially contributed to the improvements found for ASLR but not for sit and reach.

### Strength and Power Tests

Consistent with *H*_1_, the number of maximal push-up test repetitions increased from pre- to postintervention for participants in the movr group but not for participants in the control group. This is likely because of the movr app’s focus on improving muscular strength and endurance through several core, stability, mobility, and strength exercises. Specifically, bodyweight push-up exercises were prescribed during the Builder sessions. Given that participants in the movr group were found to improve their shoulder flexibility over the intervention period, it is possible that this may have also facilitated an improvement in muscular endurance, as reflected in the push-up test.

In line with *H*_2_, there were no changes in handgrip strength over the intervention period in either group. This finding is consistent with a previous 6-week yoga training RCT that found no changes in handgrip strength for the yoga or control group [41]. We are unaware of other studies measuring changes to handgrip strength following similar interventions.

Partially consistent with *H*_2_, there were significant decreases in countermovement jump height for participants in the control group from pre- to postintervention, but no changes were found for participants in the movr group. It is unclear why participants in the control group experienced this decline, but it may be in part due to the temporal aspects of the study enrollment. It is possible that the change in seasons from fall (eg, September and October) to winter (eg, November and December; wave 1 participants) and the intervention period overlapping with seasonal holidays (eg, Christmas 2019; wave 2 participants) may have reduced overall physical activity patterns and led to potential gains in body mass. For instance, holidays typically represent a time of increased weight gain associated with increased food consumption and reduced exercise [42]. Although no significant changes in body mass from pre- to postintervention were detected for either group, very slight increases were observed in participants in the control group (mean 70.45 kg to 70.77 kg) but not for those in the movr group (mean 71.22 to 71.15 kg). Even slight increases in body mass may have been enough to reduce countermovement jump flight time and subsequently led to the decreases in countermovement jump that were found.

### Cardiovascular Fitness

Partially consistent with *H*_2_, there was a significant decrease in 
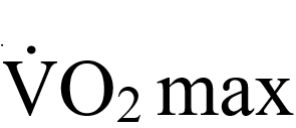
 from pre- to postintervention for participants in the control group, but no significant changes were observed for participants in the movr group. Although this was a surprising finding, it may also be explained by the aforementioned temporal factors associated with the enrollment timeline of the study. It may be that the transition from fall to winter, coupled with seasonal holidays, decreased physical activity and increased weight gain among participants. For example, outdoor forms of exercise (eg, running and cycling) would have become less accessible during winter months. Thus, these factors may have subsequently hindered cardiovascular fitness. Interestingly, although there were no significant changes in self-reported physical activity behavior from pre- to postintervention for either group, there tended to be a decrease for those in the control group (mean 2494.17 to 2210.00 MET min per week), and an increase for those in the movr group (mean 2520.70 to 2662.73 MET min per week). Another possibility is that as cardiovascular fitness levels were relatively high for all participants at baseline, there may have been less room for improvement and a greater likelihood for a potential decline over time. In any case, it appears that the tendency for a decline in 
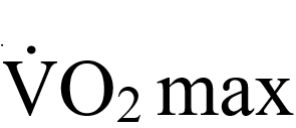
 that was apparent for those in the control group was not apparent for those randomized to the movr group. It is possible that the use of movr may have somehow mitigated these potential temporal factors that were seen for those in the control group; however, future investigation would be required to understand why this was the case.

### Practical Implications

The 100-point FMS system was developed to increase the precision of scoring and subsequently lead to greater sensitivity in detecting changes in FMS in response to interventions [13]. However, it should be noted that the use of the 100-point system is more complex to implement and requires video analysis, which may take away from the simplicity and time efficiency of using the FMS as a diagnostic tool in practice [15]. For instance, in this study, each FMS performance was carefully analyzed using two camera angles and took approximately 150 hours each for the first and second authors to fully score. This scoring system may not be feasible for practice but may be feasible for research purposes and standards.

Improvements in FMS, flexibility (eg, shoulder flexibility, ASLR, and ankle dorsiflexion), and strength (eg, push-ups) have several potential benefits and implications for musculoskeletal health and injury prevention [7,11]. For example, sufficient ankle dorsiflexion is critical for regular activities of daily living, such as walking, running, and stairclimbing, and restricted ankle dorsiflexion can contribute to overuse injuries of the foot and lower limbs [27]. Although the current findings regarding improved functional movement, flexibility, and strength are promising, future research is encouraged to determine how the magnitude of change to these outcomes found in this study may translate into specific clinically meaningful outcomes.

Importantly, we avoided incorporating any behavior change counseling or techniques into the intervention component of this study to reflect the free-living experiences of using a downloaded app. Future researchers are encouraged to explore the added benefit of incorporating theory-based behavior change techniques along with the use of the movr app. Nonetheless, the results of this study demonstrated promising initial evidence of the benefits of movr on measures of physical functioning, despite the minimalistic nature of the intervention. This may suggest that individuals can still reap meaningful benefits from the app without requiring additional counseling or resources.

To our knowledge, there are limited to no studies that have investigated the real-world impact of mHealth apps designed specifically to improve functional movement. The findings from this study provide early evidence of the potential impact similar apps may have. It also acts as a reminder of the importance of enhancing physical function and movement as a whole as a precursor and/or facilitator to improve participation in and quality of physical activity. mHealth apps aiming to increase physical activity participation may benefit from incorporating additional components that focus on enhancing functional movement.

At this time, the COVID-19 pandemic likely continues to have a significant impact on sedentary behavior and physical inactivity, and it is unclear when this will subside [16,17]. As such, digital technology can play a considerable role in curbing such trends by providing access to health and fitness alternatives in the form of mHealth. The findings of this study provide initial evidence of the effectiveness of the movr app for enhancing functional movement and physical fitness in a digital format that is highly accessible and requires minimal resources. Therefore, movr may represent a viable mHealth option for individuals as they adapt to social distancing practices.

### Strengths, Limitations, and Future Directions

This study had several strengths. As recommended in previous mHealth and technology literature [1,5], an RCT design that included a waitlist control group was implemented. We elected to use a modified research standard version of the 100-point FMS system in addition to a battery of other quantifiable flexibility, strength, and fitness assessments to allow for an interdisciplinary, comprehensive, and more sensitive assessment of functional movement and physical fitness. The intervention component of the study required minimal resources, was cost- and time-efficient, and required no additional attention from researchers. Similarly, the movr app is easy to use remotely (eg, at home, a hotel, or the gym), is highly personalized to individual needs, and only required an average time commitment of 28.94 minutes per week (11.01 min per week for Minis; 17.93 min per week for Builders) over the study period. Furthermore, this study used a pragmatic approach to capture real-world conditions. Taken together, these factors increase the ecological validity of this study. To our knowledge, this is the first randomized controlled study to evaluate the effects of an mHealth app on functional movement and physical fitness.

This study also has limitations that are worth noting. The duration of this RCT was only 8 weeks, which was sufficient to detect differences in patterns of change between conditions, but may not have been sufficient to see more drastic changes in some measures of physical fitness (eg, sit and reach, handgrip strength, and countermovement jump). Future RCTs could benefit from studying these effects over a longer period. Although participants in the movr group were ultimately free to use the app as little or as much as they wanted to on their own time, participants were aware that their app usage was being monitored. Thus, participants’ experiences using movr over the study duration may have been different from what they would have been if they had used the app outside of the study parameters. Similar to any mHealth apps, there were technical issues reported by participants over the study duration, such as the movr app crashing in the middle of a Minis or Builder session. In such cases, a workout would not have been registered as complete in the movr database; thus, it is possible that the movr usage data may have been underreported (ie, participants were using the app more than what was recorded on the movr database server). Although there was a range of activity status and age, most participants in this study were physically active, young, and healthy. Therefore, the results of the study may not generalize to individuals who are physically inactive, older, or living with a chronic disease. Future research is encouraged to determine if the current findings can be generalized to other sample populations.

### Conclusions

Although there are countless mHealth apps, very few are designed specifically to enhance functional movement and physical fitness at the individual level. movr is a novel mHealth app that uses self-reported movement patterns to prescribe workouts that cater to individual user needs. This pilot pragmatic RCT was used to empirically evaluate the movr app’s real-world impact for the first time. The findings revealed that movr improved indices of functional movement, flexibility, and muscular endurance over an 8-week period compared with the control group while maintaining handgrip strength, lower body power, and cardiovascular fitness. Taken together, this study demonstrated the potential of movr as an accessible mHealth app that may be used to enhance indices of functional movement and physical fitness.

## Appendix

Multimedia Appendix 1 Fitness testing procedures.

Multimedia Appendix 2 Sample images of fitness tests: (A) shoulder reach flexibility, (B) sit and reach, (C) active straight-leg raise, (D) handgrip strength, (E) half-kneeling dorsiflexion, (F) push-up.

Multimedia Appendix 3 CONSORT-EHEALTH checklist (V 1.6.1).

## Abbreviations

ANOVA: analysis of variance
ASLR: active straight leg raise
FMS: Functional Movement Screen
MANOVA: multivariate analysis of variance
mHealth: mobile health
RCT: randomized controlled trial
RM: repeated measures
VO_2_ max: maximal oxygen uptake
