# Supplementary material for: Examining the Impact of a Mobile Health App on Functional Movement and Physical Fitness: Pilot Pragmatic Randomized Controlled Trial
Source: JMIR Mhealth Uhealth. 2021 May 28;9(5):e24076. doi: 10.2196/24076 (PMC8196352; doi:10.2196/24076)
Supplement: Multimedia Appendix 1 [file mhealth_v9i5e24076_app1.pdf]

Multimedia Appendix 1
Fitness Testing Procedures

Note: Participants' shoes were removed and socks were worn for the performance of all fitness tests. Prior to each test, the research testers demonstrated each test to the participants while providing full instructions. Participants were asked if they understood the instructions and if they had any questions prior to commencement of each test. The shoulder reach flexibility and active straight-leg raise tests were completed as part of the seven Functional Movement Screen™ (FMS) movements in accordance with the standardized FMS verbal instructions, and were scored using a soft tape measure and a digital inclinometer, respectively.

The Sit and Reach Test

Equipment:
Sit and reach box/Flexometer

Procedure:
Participants were instructed to sit on the floor with their legs extended out straight with the soles of their feet placed flat against the wooden box. Both knees were locked and pressed flat to the floor. With their palms facing downwards and their hands on top of each other or side by side on the block, participants were instructed to reach forward along the measuring line as far as possible. After two practice attempts to ensure technique was maintained, the participants were asked to reach out and hold the end position for at least 1-2 seconds while the distance was recorded. The tester ensured sure there were no jerky movements.

Scoring:
The score was recorded to the nearest milimeter as the distance reached by the hand.

Half-Kneeling Dorsiflexion Test

Equipment:
Soft measuring tape, right-angle (90 degree) ruler, small yoga knee pad

Procedure:
Participants started in a half-kneeling stance with their back knee on a foam pad. A right-angle (90 degree) ruler was placed flush with the ground and at the position of their knee. The measuring tape was placed on the floor, extended from the end of their big toe away from their body. The participants were instructed move the knee of their vertical (front) leg forward as far as possible (creating dorsiflexion at the ankle) without lifting their heel off the ground. They were asked to hold this end position for 3 seconds while the distance was measured.

Scoring:
A right-angle (90 degree) ruler was placed flush with the ground and at the position of the knee. The measurement was taken from the big toe to the position of the knee using the measuring tape along the floor.

Handgrip Strength Test

Equipment:
Smedley spring handgrip dynamometer

Procedure:
Participants held the dynamometer in the hand to be tested, with their arm straight at their side and their elbow by the side of the body. When holding the dynamometer, the base rested on the heel of their palm with their thumb wrapped around and the handle rested on the middle of their remaining four fingers. When ready, participants squeezed the dynamometer with maximal isometric effort, which was be maintained for approximately 3-5 seconds. Participants were instructed to avoid any other body movement and to not use the side of their body to support their arm while squeezing. Participants were strongly encouraged to give a maximum effort. A total of 3 trials per side were complete, alternating right and left arms for each trial.

Scoring:
The best score from three trials for each hand was recorded, with at least 15 seconds recovery given between each trial.

Vertical Jump Test

Equipment:
My Jump 2 mobile application, iPhone 8

Procedure:
Prior to completion of the vertical jump trials, participants' straight leg length (from anterior superior iliac spine to end of big toe with ankle plantarflexed) and height at 90 degrees of knee flexion (vertical distance between the anterior superior iliac spine to the ground in a 90-degree knee-flexed position) were measured. A goniometer was used to ensure 90-degree knee flexion and a measuring tape was used to measure leg lengths. These measurements were taken as recommended by My Jump 2 and used to create participant profiles within the My Jump 2 app.

An iPhone equipped with the My Jump 2 mobile application was set up approximately 1.5m away from the participant standing spot to video record the jump trials in slow motion. The participant stood within the frame of the iPhone video camera with their legs straight and their hands on their hips. Starting from a static standing position with their legs straight, they then performed a countermovement jump (CMJ). Participants were free to go into and out of a squat position freely and were instructed to leap vertically as high as possible while keeping their hands on their hips. Once participants were in a stationary starting position with the camera ready to record, they were prompted to jump whenever they were ready and recording began. Each participant completed a total of 3 jump trials with a minimum of 15 seconds of rest between each trial.

Scoring:
Using the slow-motion videos saved to the My Jump 2 app, the testers determined the first frame in which both feet were off the ground (take-off phase) and subsequently the first frame in which at least one foot touched the ground (landing phase). The My Jump 2 application automatically calculated the vertical jump heights based primarily on the flight time (ms) of the CMJ, with estimates of velocity (m/s), force (N), and power (W) derived from this. The best vertical jump height of three attempts was reported.

Push-Up Test

Equipment:
Clear space on flat ground, small yoga knee pad

Procedure:
Participants started by setting up in a standard push-up position on the floor. They were instructed to begin on the ground with their hands, chest, and toes touching the floor, their body and legs in a straight line, feet approximately hip distance apart, their hands directly under their shoulders, and their chin aligned with the pad. Women performed a modified push-up with their knees as a pivot point as opposed to hands (according to Canadian Society for Exercise Physiology [CSEP] guidelines). Participants were asked to maintain a neutral spine and body position and to avoid dipping their hips or widening the position of their hands during the test. When test started, they were instructed to push their body off the ground until their arms were fully extended and then lower their body until their chin touched the pad. They were asked to repeat this until failure and to maintain a consistent push up tempo and avoid holding the top position of the push up.

One tester was assigned to monitor correct push up technique and the other was assigned to count the number of correct push-ups completed. If participants lost correct body position during the test, or their chin did not touch all the way down to the pad, the tester provided a verbal warning to make a correction on their next repetition. If a correction was made, the repetitions were counted. If not, the incorrect repetitions did not count.

Scoring:
The number of correctly completed push-up repetitions was recorded by the test scorer.
