# Supplementary figures and images for: Examining the Impact of a Mobile Health App on Functional Movement and Physical Fitness: Pilot Pragmatic Randomized Controlled Trial
Source: JMIR Mhealth Uhealth. 2021 May 28;9(5):e24076. doi: 10.2196/24076 (PMC8196352; doi:10.2196/24076)

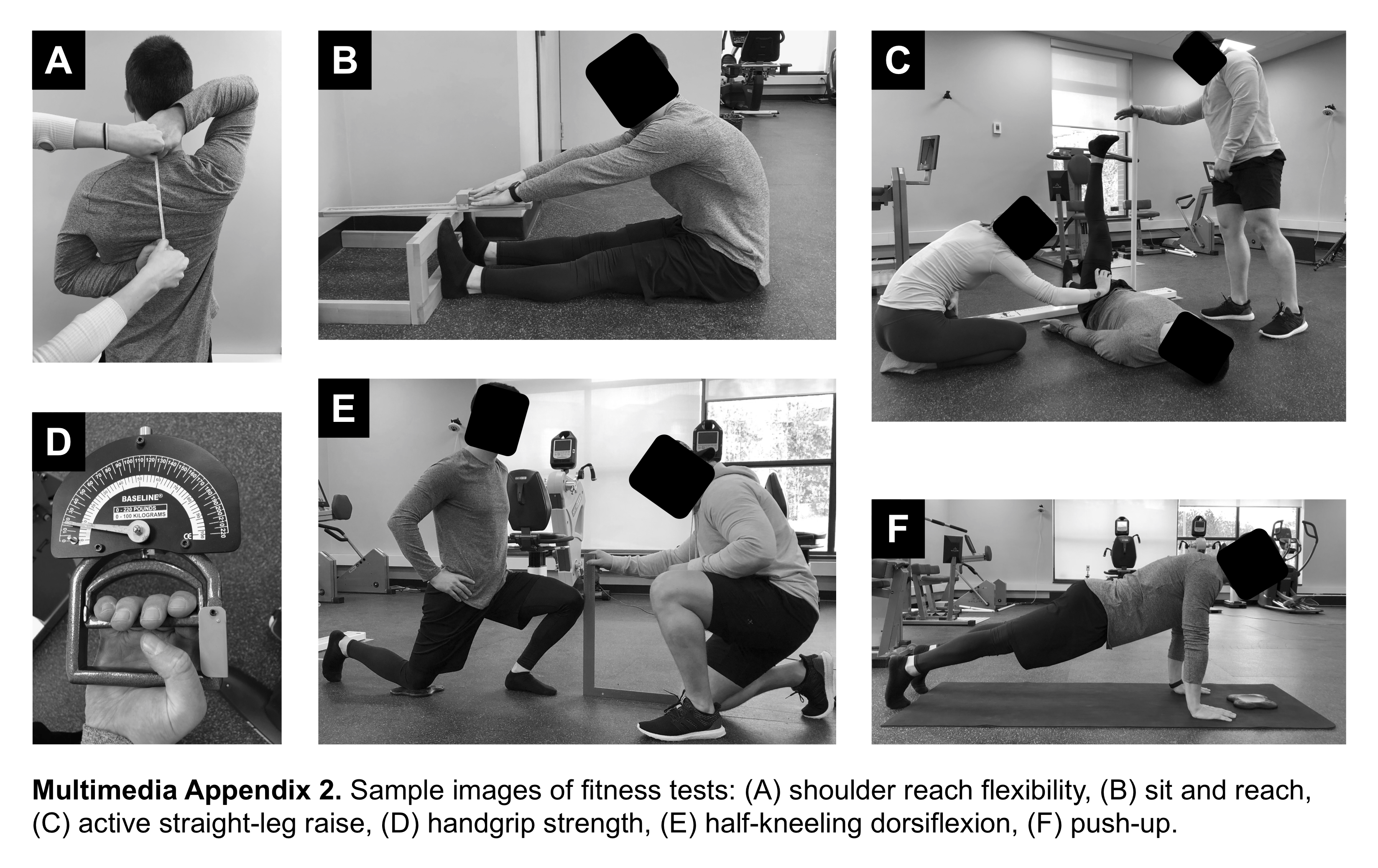

Supplement: Multimedia Appendix 2 [file mhealth_v9i5e24076_app2.png]
